# Graphene-Based Contacts for Optoelectronic Devices

**DOI:** 10.3390/mi11100919

**Published:** 2020-10-01

**Authors:** Susana Fernández, Antonio Molinero, David Sanz, José Pablo González, Marina de la Cruz, José Javier Gandía, Julio Cárabe

**Affiliations:** 1 CIEMAT, Departamento de Energía, Unidad de Energía Solar Fotovoltaica, Avda. Complutense 40, 28040 Madrid, Spain; josepablogo@yahoo.es (J.P.G.); marina.cruz@ciemat.es (M.d.l.C.); jj.gandia@ciemat.es (J.J.G.); julio.carabe@ciemat.es (J.C.); 2 CIEMAT, Departamento de Electrónica, Avda. Complutense 40, 28040 Madrid, Spain; dasago93@gmail.com

**Keywords:** graphene, ohmic contact, optoelectronic devices

## Abstract

Hybrid transparent contacts based on combinations of a transparent conductive oxide and a few graphene monolayers were developed in order to evaluate their optical and electrical performance with the main aim to use them as front contacts in optoelectronic devices. The assessment of the most suitable strategies for their fabrication was performed by testing different protocols addressing such issues as the protection of the device structure underneath, the limitation of sample temperature during the graphene-monolayer transfer process and the determination of the most suitable stacking structure. Suitable metal ohmic electrodes were also evaluated. Among a number of options tested, the metal contact based on Ti + Ag showed the highest reproducibility and the lowest contact resistivity. Finally, with the objective of extracting the current generated from optoelectronic devices to the output pins of an external package, focusing on a near future commercial application, the electrical properties of the connections made with an ultrasonic bonding machine (sonic welding) between the optimized Ti + Ag metal contacts and Al or Au micro-wires were also evaluated. All these results have an enormous potential as hybrid electrodes based on graphene to be used in novel designs of a future generation of optoelectronic devices, such as solar cells.

## 1. Introduction

Although graphite, one of the crystallographic forms of carbon, has been relatively well known for a long time, its two-dimensional version—graphene—is a much more recent object of research. The lamellar structures of graphite and of thermally reduced graphite oxide were already known in the mid-19th century [1], but it still took about ninety years until the first theoretical studies on single atomic graphite layers were undertaken and the first electron-microscopy images of a few-layer graphite sample were published [2]. Despite these partial milestones, the inception of graphene technology had to wait another half century before André Geim and Kostya Novoselov at the University of Manchester were able to obtain single-atom layers from a graphite sample in 2004 [3]. The method is based on peeling single graphite layers and transferring them onto a monocrystalline-silicon wafer coated with silicon dioxide by following a procedure called micromechanical cleavage.

Graphene is defined as the material made of a single layer of carbon atoms distributed in the vertexes of a hexagonal network. This hexagonal shape (honeycomb) results from the covalent bonds generated by the overlap of the sp^2^ hybrid orbitals of the mutually-bonded carbon atoms. It is therefore a two-dimensional material (2D). Such an atomic arrangement and its two-dimensional structure are the factors conferring extraordinary properties to graphene.

Graphene is also a zero-gap semiconductor. Its electron mobility is extremely high, of the order of 150,000–200,000 cm^2^⋅V^−1^⋅s^−1^. A single layer of graphene absorbs 2.3% of incident light, allowing around 97.7% to pass through [4]. As a rule of thumb, when working with graphene multilayers, one can estimate a 2.3% transmittance loss per atomic layer. The degree of reflection from single-layer graphene is almost negligible, just less than 0.1%, rising to no more than ≈2% for ten atomic layers [5]. Finally, graphene is not a self-standing material. Once deposited or obtained by whatever technique, it must be transferred onto some kind of substrate conferring to it the necessary mechanical stability among other conditions. The sensitivity of graphene and of its charge-conduction mechanisms to the interaction with the substrate or any other adjacent substance is not at all negligible, so the choice of any material to be combined with graphene implies careful consideration from the mechanical and electrical points of view.

Many of the described properties of this new material are outstanding in many senses, but for the purpose of the present work, the above-mentioned electrical and optical features are particularly relevant. The possibility of obtaining an excellent sheet conductance in combination with a very low absorption of light (for one or a few atomic layers), added to an extremely low reflection to visible light, all of this without adding more than a few angstrom to device thickness, makes graphene an excellent candidate for those optoelectronic applications in which low sheet resistances, exceptional responses at high frequencies, low contact resistances or reduced light absorption are required. A wide range of optoelectronic devices such as light emitting diodes, photodiodes, liquid-crystal displays, touch screens or solar cells require transparent or semi-transparent contacts having such properties [6]. In addition, many of these optoelectronic devices typically use transparent conductive oxides (TCOs), such as fluorine doped tin oxide (SnO_2_:F) abbreviated as FTO, indium tin oxide (In_2_O_3_:SnO_2_) abbreviated as ITO, and aluminum doped zinc oxide (ZnO:Al_2_O_3_), abbreviated as AZO. They usually play the role as transparent electrodes, and thus, they must have n-type conductivity [7]. The TCOs are fabricated by using a wide variety of methods such as chemical vapor deposition (CVD), magnetron sputtering, sol–gel process or spray pyrolysis [8]. Among them, although magnetron sputtering can be considered too expensive for some industrial applications, it has two special advantages: (i) its great scalability to large areas, and (ii) its easiness to achieve thin films with good performance even if fabricated outside its thermodynamic equilibrium. Both facts are relatively important to make a success of the incorporation of the thin films into the low-temperature device technology [9]. For these reasons, in the present work, magnetron sputtering is chosen to fabricate the TCO materials.

Among the TCO materials, ITO is currently the most commonly used in silicon heterojunction solar cell (SHJ) technology. This semiconductor material is well-known from its mass production and exhibits very suitable optoelectronic properties. Generally, ITO is deposited by direct current (DC) magnetron sputtering on large areas [10]. Otherwise, AZO is also used in the SHJ technology. AZO is one of the very few alternative candidates for the replacement of indium based TCOs. This is due to its low-cost, its abundance, and its chemical stability in hydrogen plasma, in comparison with other TCOs [11]. These well-known materials have a reasonable transmittance >80% in the visible region of the spectrum [12]. In certain applications requiring limited TCO thicknesses, such as SHJ front contacts, sheet resistances are not lower than 100–120 Ohm/sq [13]. These front contacts play an important role for SHJ to achieve both efficient carrier collection and transport properties. Furthermore, contact resistances and optical reflectances would contribute to better device performances if they were improved (i.e. reduced). In this sense, hybrid concepts combining various kinds of nanoscale materials have been attempted. For example, the incorporation of carbon nanotubes (CNT) [14], metal nanowires/nanogrids [15,16], graphene or reduced graphene oxide (rGO) [17,18] have already been demonstrated, showing successful results. Among them, sheet resistances of 24 Ω/sq and 83% of average transmittance have been reported by CNT-based electrodes [14], while using graphene fabricated by chemical vapor deposition (CVD), a wide range of values are found depending on the transfer techniques [19]. In all these cases, the graphene replaces the TCO commonly used. However, in SHJ technology, the TCO plays an important role as anti-reflective coating and hence, it cannot be avoided. Therefore, hybrid graphene/TCO structures capable of enhancing the performance of the SHJ technology are highly desirable for that particular application. This research continues at the early stage of development because currently, the most common role for graphene is the TCO substitution, not a combination with it, as is shown in this work. With this concept in mind, the TCOs fabricated in this research are the most commonly used in SHJ technology, namely, ITO and AZO. The main reason for this choice is to avoid deviating too much from the materials used industrially [10]. 

On the other hand, to efficiently extract the current in the SHJ device, a front metal grid must be evaporated finally. Therefore, it is essential to achieve low resistive metal contacts. As the graphene atomic layers are part of the hybrid electrode concept, appropriated metal stacks with ohmic character deposited on top of graphene also play a vital role in the device operation. In graphene-based technology, several investigations have been devoted to check different metals with proper work functions for electronic devices such as field-effect transistors [20]. These studies are based on modifying the Fermi-level difference between metal and graphene in order to improve the metal/graphene interface. One of the best results has been achieved with Pd contacts showing contact resistivities close to 200 Ωµm at carrier concentrations of 10^13^ cm^−2^. Tests involving different metals, surface treatments and innovative architectures have also been reported. Among them, Cr/Pd/Au (1/15/50 nm) metal stack fabricated by e-beam evaporation on CVD graphene using a pre-plasma treatment presenting ~270 Ωµm contact resistivity [21], Ni/Au (25/50 nm) stack fabricated by evaporation on CVD-graphene showing ~300 Ωµm contact resistivity [22] or Pd/Au (5/50 nm) stack fabricated by e-beam evaporation on exfoliated graphene reaching a ~69 Ωµm contact resistivity [23]. Regarding SHJ technology, the main metals used can be Ti/Pd/Ag, Ti/Cu, Ti/Al, Al [24,25] or Ti/Ag [26]. As can be observed, the Ti layer is always combined with a stack of metals to obtain a minimal finger resistivity due to the poor conductivity of this metal. In addition, in this technology, thermal evaporation is preferred because it does not degrade the quality of the passivation layers [24]. Therefore, in our case, the main metals tested are Ti, Al, Ag and Pd.

The main aim of this work is to find a suitable combination of graphene and any of the available transparent conductive oxides showing an appropriate performance for it, to be applied to make electrodes for optoelectronic devices such as solar cells. The evaluation of preparation conditions compatible with the integrity of the properties of both materials has been treated as a key issue. In addition, considering that one of the major limitations to the incorporation of graphene to such devices is the contact resistance arising at the metal/graphene interface, the optimization of the metal-stack combination suitable for the solar device has also been addressed. With the challenge of developing devices based on hybrid graphene/TCO-based transparent contacts for commercial applications, additional tests about how the sonic welding of aluminum (Al) or gold (Au) micro-wires to Ti + Ag front contacts could modify the contact resistance of the metal/graphene interface were also carried out. At this stage, the ideal Ti + Ag thickness layer and the setting of the parameters used in the semiautomatic wedge-wedge bonding machine were calculated to minimize the resistance of the sonic welding and to avoid interface damage. The results derived from this investigation have a considerable potential for the development of advanced transparent or semitransparent electrodes based on graphene and its application in novel designs of optoelectronic devices. The research of such a combination and of adequate preparation protocols is the kernel of the present paper.

## 2. Materials and Methods 

### 2.1. Graphene-based Transparent Contact Fabrication

The hybrid transparent contacts described in this work are based on the combination of a common TCO and 1 to 3 graphene monolayers. In most optoelectronic devices, an ohmic low-contact-resistance metal electrode is essential in order to extract current with the lowest power losses. Hence, this metal stack plays a relevant role in device operation. The choice of the metallization is a critical step in outlining the cost and efficiency of solar cells and is considered as a vital factor for achieving high efficiencies. For this reason, strategies to reduce the metal contact resistance were also developed in this work. 

The graphene monolayers were grown by the Spanish company Graphenea S.L. by chemical vapor deposition (CVD) on copper foil from CH_4_ precursor, prepped for transfer with polymethyl methacrylate (PMMA) coating, and finally transferred to the desired substrate. The CVD fabrication technique was preferred because it can produce relatively high-quality and high-purity graphene and potentially on a large scale. More details about the graphene fabrication can be found in reference [27].

The TCO materials were fabricated using a commercial Leybold UNIVEX 450B magnetron sputtering system (Leybold GmbH, Cologne, Germany). Aluminum-doped zinc oxide (AZO) was deposited by using a ceramic 4-inch ZnO:Al_2_O_3_ (98/2 wt.%, Neyco, Vanves, France) commercial target subject to radio-frequency (RF), and indium tin oxide (ITO) was obtained from a ceramic 4-inch In_2_O_3_:SnO_2_ (90/10 wt.%, Neyco) commercial target subject to direct current (DC). Argon of a purity of 99.999% was used as the inert gas in the sputtering process, and the control of its flux was carried out by means of a mass-flow controller. All the TCO films were deposited at low temperature in order to work in conditions compatible with the preparation of different kinds of optoelectronic devices having a particular sensitivity to high temperatures.

Different combinations of TCO and graphene were tested, with different deposition and transfer sequences in order to assess technological paths taking into account the mutual compatibility of the processes involved as well as the properties of the resulting multilayers.

Three different substrates were used: 1 × 1 cm^2^ quartz to make electrical measurements, 2 cm × 2 cm Corning glass to produce samples for testing transparency, and 2 cm × 2 cm silicon substrates yielding samples suitable for the assessment of their antireflecting capability. The latter is considered an essential property in the specific case of front transparent contacts for silicon-based solar cells.

The metal stacks were evaporated by thermal evaporation on the graphene-based transparent contacts by using a commercial UNIVEX 300 system. The metals tested in the different schemes were aluminum (Al), palladium (Pd), nickel (Ni), titanium (Ti) and silver (Ag), respectively. This choice was done based on metal costs, except for Pd, which was used because it is one of the most commonly used metals in high-efficiency photovoltaic device technology. 

### 2.2. Graphene-based Transparent Contact: Characterization Techniques 

**Figure 1:**
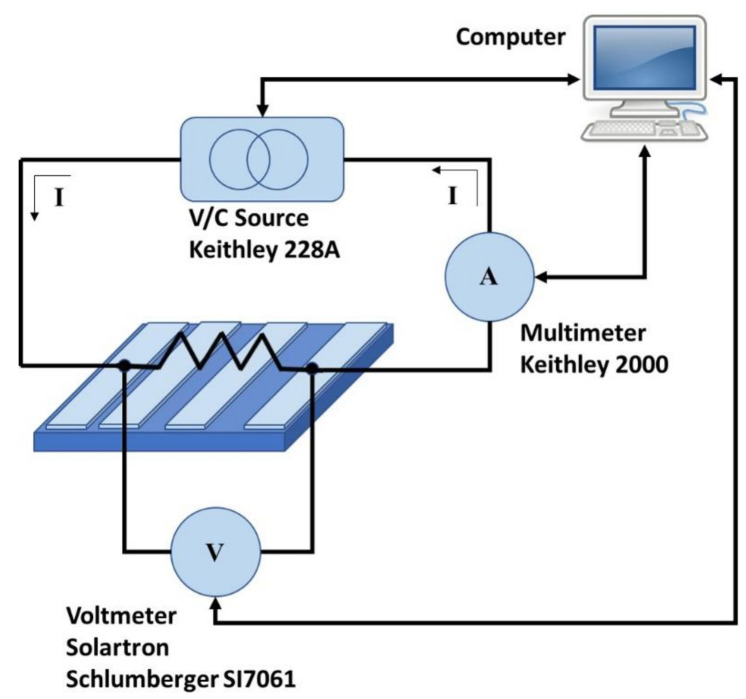
Scheme of the system used to determine the electrical parameters in this work.

To determine the electrical parameters, four metal coplanar parallel electrodes of 0.1 cm width and 1 cm length unequally spaced (0.0366 cm, 0.0864 cm and 0.2379 cm, respectively) were deposited by thermal evaporation onto the samples. These monolayers had been previously transferred onto 1 cm × 1 cm quartz substrates. These measurements were carried out by testing 4-point electrical resistance between all the possible electrode pairs with two contacts on each electrode. The electrodes were contacted by means of 4 micro-positioners, a power supply was used to bias the samples, an electrometer measured currents and a voltmeter provided voltages. The overall system is pictured in Figure 1. Parameters such as the contact resistivity (*ρ_c_*) and the current transfer length (*L_T_*) were determined for the metal stacks, as well as the sheet resistance (*R_s_*) of the transparent contacts, by using the transmission line method (TLM) [28]. These TLM structures involved metal contacts fabricated on the diffused conducted graphene regions. Current-voltage (I-V) measurements on contacts of particular dimensions and varying the spacing between them were measured to obtain the total resistance *R_T_*. Then, *R_T_* was plotted as function of the contact spacing d to extract parameters such as the *L_T_*_,_ measured from the intersection of the *R_T_* curve for *R_T_* = 0, and the ρ_c_, usually obtained from the plot using the extracted *L_T_* and the sheet resistance *R_s_* [29].

**Figure 2:**
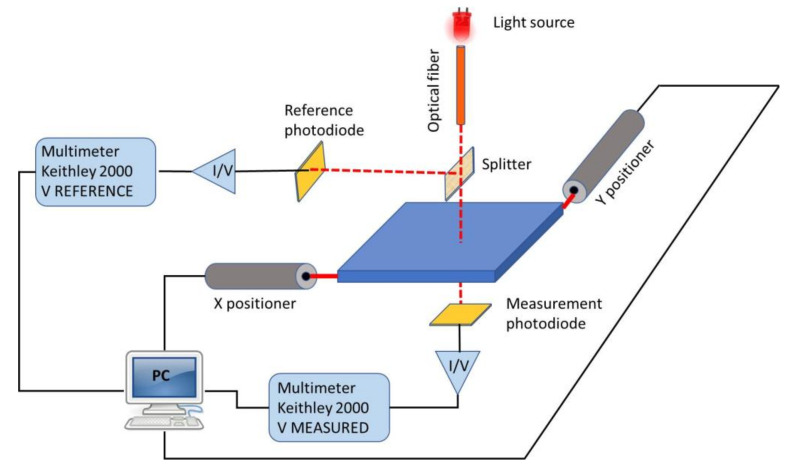
Scheme of the home-made system to determine the optical parameters in this work.

To determine the fraction of light transmitted by the different layers that compose the transparent contact, optical transmission maps were obtained by using an experimental optical-transmittance-mapping (OTM) system that combines a focused white-light lamp, an X-Y linear-positioner set, a pair of current pre-amplifiers, a reference photodiode and a pair of digital voltmeters. Figure 2 shows a picture of the OTM system. It is controlled by specific software that produces an image where each point registered is associated with a transmittance value. This characterization technique can result very useful to determine the optical homogeneity of the transparent contact before incorporating it into the final device, as well as to check the number of graphene monolayers present in the sample.

Finally, the optical total (hemispherical) reflectance of the transparent contact as function of the wavelength was measured using a UV/Visible/NIR Perkin Elmer Lambda 1050 spectrophotometer, equipped with a 6-mm-diameter integrated sphere.

### 2.3. Wire Bonding: Processing and Characterization

The ultrasonic wire bonding technique is considered as one of the most flexible and cost-effective to assemble the vast majority of semiconductor packages. It commonly uses either Au or Al wires with tens of micrometers of diameter to interconnect integrated circuits (IC) or other semiconductor devices, and to package during the semiconductor device fabrication. Hence, it can be very useful to connect an IC to other electronics, or to connect from one printed circuit board (PCB) to another.

**Figure 3:**
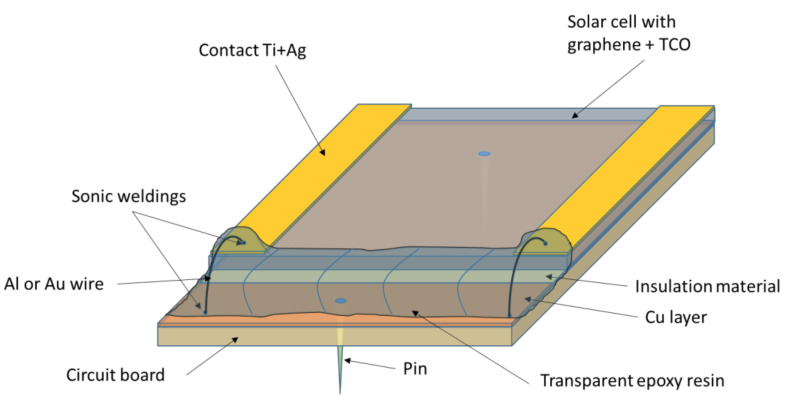
Sketch of a solar cell with a hybrid graphene/ transparent conductive oxides (TCO)-based transparent contact and Au or Al micro-wires sonic welded.

To extract the current generated by the optoelectronic device is mandatory to interconnect the optoelectronic device to the supporting board, as it is depicted in Figure 3.

In this work, the ultrasonic wire bonding technique (labeled as sonic welding) was chosen to make the connections. For this purpose, the bonding machine used was the 5632DA model from F&S BONDTEC Semiconductor GmbH (Braunau am Inn, Austria) [30]. Three main parameters must be set in this machine to minimize the connection’s resistance: force, power and time. “Force” defines the mechanical force that must be applied to the connection during welding; “power”, the energy per time unit applied by the ultrasound during welding; and “time” sets the welding time. For this study, a force of 32 cN was applied, an ultrasonic frequency of 67 kHz and 29 ms as welding time. At this early stage of development, 25 µm-size diameter AlSi (1%) wires were tested to connect the Ti + Ag front contact of the optoelectronic device to the copper (Cu) pad on the PCB. Hence, two connections must be made: the first one, the AlSi (1%) wire to the Ti + Ag contact, and the second one, the AlSi (1%) wire to the Cu pad. The resistances of the AlSi (1%) wire and the two connections should be added to the metal/graphene interface contact resistance to create a more realistic scenario.

**Figure 4:**
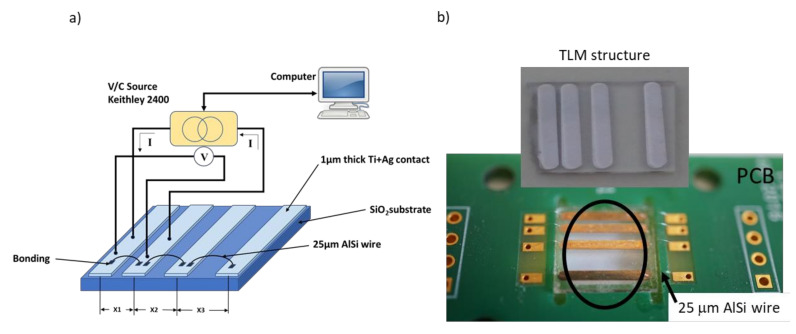
(**a**) Scheme of the home-made system for transmission line method (TLM) method used to calculate the contact resistances after the wire bonding, and (**b**) photograph of the sample connected to the PCB with the AlSi wires.

To measure sonic welding contact resistance, a four-wire technique was used with the home-made system pictures in Figure 4a. A Keythley 2400 I/V source/meter was used to source current and to measure voltage in different pad pairs. The TLM method was used to study only the DC properties [31] (for this specific application, the AC connection properties are not of interest). Four Ti + Ag pads were placed at different distances (X1, X2, X3), and they were interconnected through AlSi wires, welded with the ultrasonic bonding machine. From resistance values calculated for each pad pair, the welding contact resistances were calculated. Figure 4b shows a photograph of a TLM structure fabricated to be connected to the PCB with AlSi wires to determine the contact resistance. 

## 3. Results and Discussion

### 3.1. Optimization of the Metal Stack on Graphene Layers

The contact resistivity ρ_c_ of the conventional metal stacks previously described was determined for the different metal stacks tested. The metal stacks were evaporated at room temperature on graphene monolayers, bilayers and trilayers, respectively, previously transferred onto quartz substrates at the common transfer process conditions used by the supplier of the graphene monolayers (www.graphenea.com) [27]. The metal combinations in the study were Al, Ni + Al, Pd and Ti + Ag. The contacts based on large-work-function metals as Pd, Ni, and Ag were expected to provide low contact resistance due to their small reactivity compared to other small work function metals [32]. Al and Pd were used pure, with thicknesses of 200 and 50 nm, respectively. Ni and Ti were used in combination with the top Al and Ag layers, respectively, deposited in the same evaporation step. The main role of Al and Ag was to reduce the series resistance of the contacts. The thickness combination used was 20/200 nm in both cases. These metal stacks were chosen because they are those most used in photovoltaic technology. 

Table 1 summarizes the main results obtained for the contact resistivity *ρ_c_* and transfer length *L_T_* depending on the metal stack used. In addition, the sheet resistance *R_s_* of the graphene material underneath was estimated in each case.

It was found that the contact resistivity to graphene monolayers was lower if using Ti + Ag and Ni + Al metallic combinations. Pd and Al contacts were much more resistive. The sheet-resistance data obtained by the graphene monolayer using Al for the contact were very disparate, as seen in the out-of-range value of 843 Ω/sq and its wide error bar. This value was far from that given by the supplier [27] and those found in the literature [33]. This deterioration observed in the sheet resistance of the graphene atomic layers was probably a consequence of Al diffusion into graphene material leading to the formation of other compounds such as aluminum carbide. This formation would affect the graphene order and would lead to a deterioration of electrical properties, as other authors described [34].

From the results in Table 1, it can be observed that the lowest contact resistivity obtained was that for Ni + Al tested on the graphene monolayer. However, very poor data reproducibility was achieved with this metal stack. This fact was attributed to the hypothesis of Ni not being able to be an effective seed layer, probably owing to its non-uniform thickness or to edge effects, leading to an irregular and unpredictable Al diffusion into graphene. At this stage, only two metallic combinations, Ti + Ag and pure Pd were tested on three graphene monolayers. The slightly smaller contact resistivity of the 20/200 nm thick Ti + Ag stack compared to the 50 nm thick Pd could be attributed to a lack of adherence to the latter and to its highly non-uniform surface coverage [32]. Regarding the current transfer length L_T_ calculated, the longest value was reached by the Ti + Ag contacts. That implies that electrons travel a longer distance in the graphene beneath the contact before flowing up into it. A possible explanation about why the metal stack based on Ti presents a better electrical performance could be related to the positive difference of the work function at the graphene/Ti interface, close to 0.15 eV [35]. A charge transfer would be occurring between Ti and graphene to align the Fermi-levels. This might result in an effective n-type doping of the graphene material and hence, in an improvement of electrical contact parameters, as it was obtained.

### 3.2. Hybrid Graphene/TCO Transparent Contacts 

#### 3.2.1. Evaluation of the Compatibility of the Preparation Conditions

The compatibility between the conditions used in the graphene transfer method (chemical reagents, temperature in vacuum and in inert atmosphere, pressure or activation conditions in different environment, among others) and the temperature applied to fabricate the cell material was evaluated. It is very important to perform these analyses because the hybrid transparent contact based on graphene is incorporated at the last manufacturing step. Hence, parameters such as the temperature and the environment used in the transfer process could negatively affect the performance of the material located underneath and that constitutes the device. For this reason, low- and high-temperature transfer procedures were mutually compared; avoiding O_2_ plasmas and UV-O_3_ gentle activation steps. The high temperature regime includes processing steps at a temperature range from 200 to 450 °C, that is, annealing in vacuum at 200 °C, the use of temperature up 450 °C in inert atmosphere, or both; while the low temperature regime avoids those steps and the temperature is not superior to 120 °C [27]. Table 2 shows the average white-light transmission, measured with the system pictured in Figure 2, and sheet resistance *R_s_*, measured with the system presented in Figure 1, for graphene samples of different thicknesses transferred at high and low temperatures, respectively. For comparison, the electrical data given by the supplier, Graphenea S.L., are included. In this case, the transfer was carried out using standard parameters on SiO_2_/Si substrates [27].

It can be noticed that the average white-light transmission values were in agreement with the theoretical predictions made according to the number of graphene monolayers [36]. Therefore, that parameter was not affected by the temperature used during the transfer process. In the case of the sheet resistance *R_s_*, a slight electrical improvement was observed in the graphene material transferred at low temperature. This work undergone so far clearly suggests the possibility of reducing the temperature in that process while the material performance is maintained. In addition, the low substrate temperature would also benefit the deposition of the hybrid transparent contacts based on a TCO by reducing the possible damage to it.

The second step consisted of evaluating the position where the graphene monolayers should be placed within the hybrid transparent contact structure. This required the study of the possible damage to graphene material produced by the bombardment of the high-energy sputtered particles during the TCO deposition. For that, three graphene monolayers were placed in two positions: (i) transferred onto the already TCO-coated resistive glass (structure 1, in Table 3) and (ii) transferred onto the quartz substrate where the TCO was subsequently sputtered on them (structure 2, in Table 3. The TCO used was 40-nm thick ITO thin film, and to compare both structures, the graphene transfer process on quartz and on the TCO-coated resistive glass was performed at low temperature. Table 3 shows the optoelectronic properties obtained for both structures in study.

These data revealed better optoelectronic properties for structure 1 where the graphene was transferred onto the ITO coated glass. These results confirm the potential damage on graphene caused by the sputtering deposition leading to a worsening of the transparent-contact performance [37].

#### 3.2.2. Performance of the Hybrid Transparent Contacts 

Taking the results described above into account, the hybrid transparent contacts were fabricated using structure 1 (the graphene transferred on top of a TCO-coated substrate) where the graphene material was transferred at low temperature. AZO and ITO materials were incorporated into the contact to enhance both the optical and electrical device performance. From the optical point of view, the main role of the TCO is to act as an antireflection coating diminishing the amount of light reflected on the solar-cell surface; that means, to eliminate unwanted reflection and increase the overall transparency. Hence, the thickness of the TCO must be fitted so that the wavelength in the dielectric material is one quarter the wavelength of the incoming wave, according to formula (1)

(1) $$nd=\frac{\lambda }{4},$$

where n is its refractive index, d is its thickness and λ is the wavelength. For photovoltaic applications, thickness is chosen in order to minimize reflection for a wavelength of 0.6 µm. This wavelength is chosen because it is close to the peak power of the solar spectrum [38]. Therefore, the thickness of AZO and ITO was close to 80 nm.

From the electrical point of view, the TCO must present the lowest possible resistivity to reduce device resistance and to extract the current in the most efficient way. Under these considerations, the performance of the hybrid transparent contacts was evaluated from the figure of merit (FOM) ϕ proposed by Haacke [39] given by the following formula (2):

(2) $$\phi =\;T^{10}/R_{s},$$

where T is the average optical transmittance and $R_{s}$ is the sheet resistance of the films.

**Figure 5:**
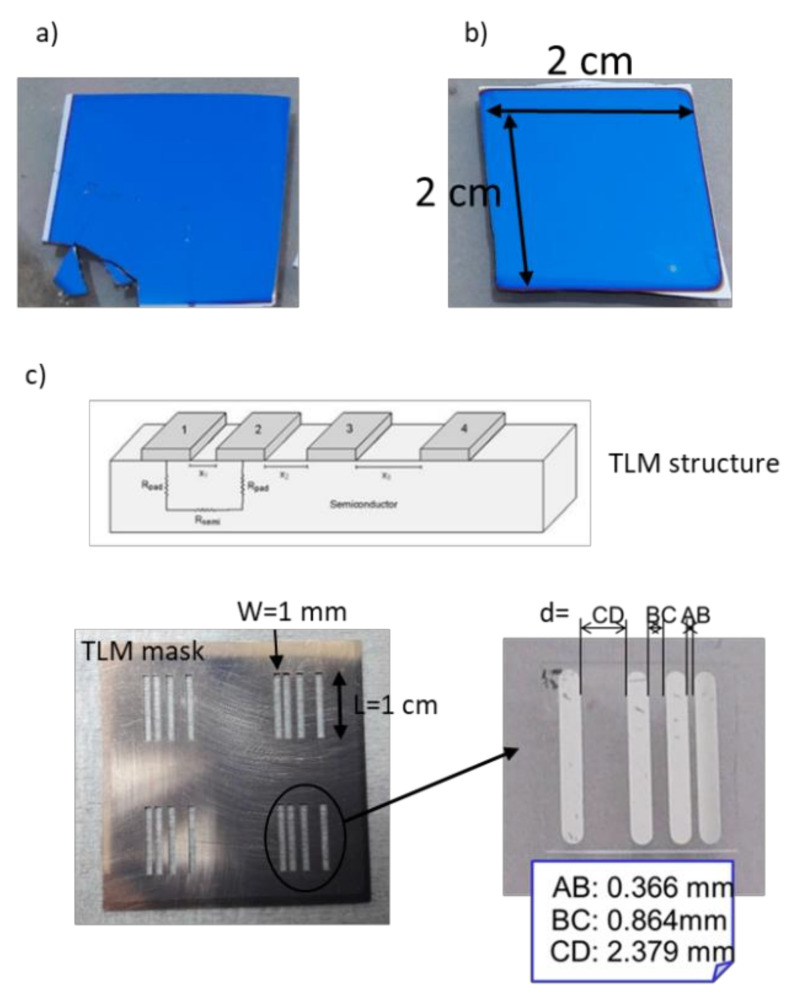
Photographs of hybrid transparent contacts based on TCO-coated silicon and (**a**) a graphene monolayer, (**b**) graphene bilayer on top and (**c**) TLM mask (on the left) and the TLM structure fabricated on graphene monolayer transferred onto quartz (on the right).

Figure 5 shows a photograph of the graphene monolayer (Figure 5a), graphene bilayer (Figure 5b) both transferred at low temperature on 2 × 2 cm^2^-size TCO-coated silicon wafers, and the TLM mask used in this work with the further TLM structure based on Ti + Ag evaporated on graphene monolayer transferred onto quartz (Figure 5c) to extract the electrical parameters.

Table 4 summarizes the optoelectronic results achieved by the different hybrid transparent contacts fabricated in this work, where one, two and three graphene monolayers are incorporated with ITO and AZO, respectively.

It can be noticed that the sheet resistance *R_s_* of the hybrid transparent contact clearly depends on the TCO material used. A detriment in *R_s_* was obtained when AZO was used. This may be attributed to a possible diffusion of the Al dopant from AZO occurring in the whole structure, which would negatively affect the electrical performance of the graphene monolayer located at the graphene/AZO interface [33,40,41]. For this reason, the sheet resistance *R_s_* of the hybrid contact obtained with three graphene monolayers transferred on top was clearly improved, compared to that one that incorporated two monolayers. In any case, the *R_s_* of the AZO-based hybrid transparent contact was not much lower than the *R_s_* of the bare 80 nm-thick AZO layer of 120 ± 10 Ω/sq. 

**Figure 6:**
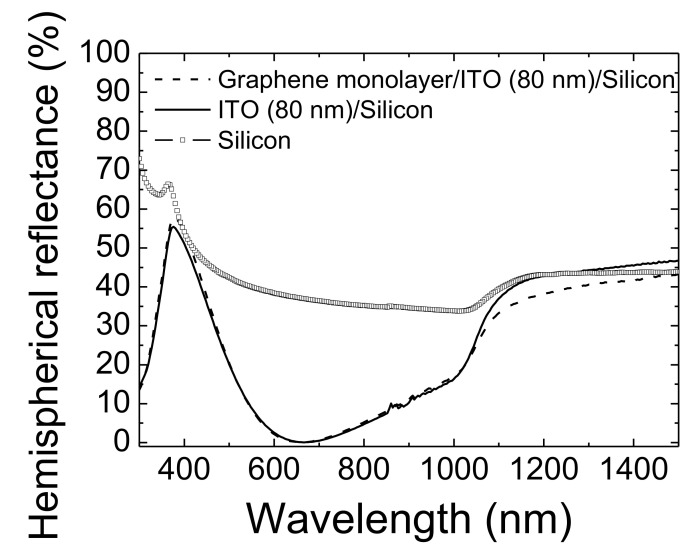
Total (hemispherical) reflectance spectra of hybrid transparent contact based on 80 nm-thick ITO layer coated silicon and a graphene monolayer on top (dashed line), 80 nm-thick ITO layer deposited on silicon (solid line) and the bare silicon (square symbols).

On the other hand, a different electrical behavior was achieved when ITO was incorporated into the hybrid transparent contact. No deterioration was observed in *R_s_* in comparison with the *R_s_* value of bare 80 nm-thick ITO layer of 85 ± 5 Ω/sq. Hence, it may be assumed that no damage would take place at the graphene/ITO interface. Finally, the best FOM value was achieved when ITO was incorporated into the hybrid transparent contact, even incorporating only one graphene monolayer. Hence, the hybrid contact based on ITO and one monolayer presented the best optoelectronic performance. Finally, Figure 6 shows the total (hemispherical) reflectance spectrum of this hybrid transparent contact as a measure of its antireflectance (AR) capability. For comparison, the spectra of the bare silicon and of the 80 nm-thick ITO layer deposited on silicon are also included.

The results indicate that the average hemispherical reflectance at the wavelength range of 400–1000 nm was 12.2% in the case of the hybrid transparent contact and slightly lower (11.7%) for the bare ITO layer. This almost negligible difference was considered within the measurement error; therefore, the addition of the graphene material into the transparent contact would not affect its AR capability. 

#### 3.2.3. Measurement of the Wire Bonding Resistance 

With the future goal of integrating the optoelectronic device based on the optimized hybrid graphene/TCO transparent electrode described above into a PCB, it is necessary to minimize the resistance connections of the wire bonding used. For this reason, the contact resistance of the welding Ti + Ag metal contact-AlSi wire, made with the ultrasonic wire bonding machine showed in Section 2.3, was measured by using the TLM method as shown in Figure 4. The contact resistance of 25 µm-thick AlSi wire calculated over three samples with Ti + Ag metal contacts deposited on a SiO_2_ substrate were around 40 ± 12 mΩ. These values are found to be typical for sonic welding at ambient temperatures using AlSi wires and Ag substrates; this is a very promising result for the applications of the graphene-based technology. So far, no tests have been done on solar cells with the hybrid transparent electrodes, but they are foreseen in the near future. In depth studies about damage produced by the wire bonding on graphene/metal interface should be done.

## 4. Conclusions

This work presents the optimization of a metal stack combination being a candidate to make ohmic contacts for silicon-based photovoltaic devices. The metal combinations tested were Ti + Ag, Al, Ni + Al and Pd, being the first one that showed the most reliable and reproducible data with the lowest specific contact-resistance value.

The evaluation of the preparation protocols used in the fabrication of the hybrid transparent contacts based on a common TCO and graphene monolayers was also studied. The first parameter evaluated was the temperature used during the graphene transfer process. Reduced sheet-resistance values of the graphene monolayers were obtained at low temperature. The optimum spatial configuration of the layers forming the hybrid transparent contacts was also determined. A strong effect on electrical properties of graphene due to the bombardment of the highly-sputtered atoms during the sputtering process of the TCO thin film was observed. Hence, adequate preparation conditions should include transferring the graphene monolayers onto the TCO and not the opposite. A correlation between the avoidance of aluminum (both in the metal contact and in the TCO) and good and repeatable electrical properties was found. Combinations of graphene with ITO were systematically better than those of graphene and AZO.

Therefore, a suitable combination of graphene and ITO thin film in a hybrid transparent contact deposited on silicon was found, showing appropriate performance for it to be used as an electrode in optoelectronic devices, more specifically, for silicon-heterojunction solar cells. Finally, 25 µm-thick AlSi bonding wires were used to connect the samples with Ti + Ag contacts to a PCB. The contact resistances of such wires were around 40 ± 12 mΩ, resulting in suitable values for this type of wire bonding at ambient temperature.

All these approaches have an enormous potential to open new horizons to achieve the definitive take-off of graphene technologies for electrical standard and transparent contacts.

## Figures and Tables

**Table 1:** Contact resistivity *ρ_c_*, current transfer length *L_T_* and sheet resistance *R_s_* of graphene atomic layers determined for the different metal stacks tested on graphene monolayer (1) and graphene trilayer (3), respectively.

Metal stack	Ti + Ag	Al	Ni + Al	Pd
*R_s_* (Ω/sq) (1)	399 ± 4	843 ± 362 (*)	383 ± 16 (*)	328 ± 12
*ρ_c_* (Ω-cm^2^)	(2.6 ± 0.5) × 10^−4^	(4.1 ± 0.3) × 10^−1^	(2.1 ± 0.7) × 10^−4^	(1.50 ± 0.04) × 10^−1^
*L_T_* (cm)	(2.80 ± 0.03) × 10^−3^	(2.57 ± 0.01) × 10^−2^	(1.80 ± 0.05) × 10^−3^	(2.14 ± 0.08) × 10^−3^
*R_s_* (Ω/sq) (3)	120 ± 5	-	-	120 ± 11
*ρ_c_* (Ω-cm^2^)	(6.5 ± 3) × 10^−3^	-	-	(4.30 ± 0.08) × 10^−2^
*L_T_* (cm)	(7.3 ± 0.5) × 10^−3^	-	-	(3.6 ± 0.8) × 10^−4^

(*) Poorly reproducible data.

**Table 2:** Average white-light transmission and sheet resistance *R_s_* for graphene samples of different thicknesses transferred at high and low temperatures, respectively. The nominal *R_s_* data of graphene materials transferred on SiO_2_/Si provided by Graphenea (www.graphenea.com) are included for comparison.

Number of Graphene Monolayers	Average White-light Transmission (%)	*R_s_* (Ω/sq): Transferred at High Temperature on Resistive Glass	*R_s_* (Ω/sq): Transferred at Low Temperature on Resistive Glass	Nominal *R_s_* (Ω/sq): Transferred at Standard Conditions on SiO_2_/Si
1	97.6 ± 1.0	375 ± 25	295 ± 25	350 ± 50
2	95.7 ± 1.0	175 ± 10	155 ± 10	188 ± 3
3	93.3 ± 0.9	120 ± 15	125 ± 15	126 ± 6

**Table 3:** Sheet resistance *R_s_* and average white-light transmission for the structures in the study where the graphene trilayer is located in different positions. The optoelectronic data of the indium tin oxide (ITO) thin film and the graphene trilayer transferred on quartz are included for comparison.

Sample	*R_s_* (Ω/sq)	Average White-light Transmission (%)
ITO (40 nm)/Glass	196.5 ± 1.0	89 ± 5
3 Graphene monolayers/Quartz	120.0 ± 5.0	93 ± 2
Structure 1: 3 Graphene monolayers/ITO (40 nm)/Glass	156.5 ± 15	80 ± 6
Structure 2: ITO (40 nm)/3 graphene monolayers/Quartz	200.0 ± 34.0	78 ± 4

**Table 4:** Sheet resistance *R_s_*, average white-light transmission and figure of merit calculated for the hybrid transparent contacts in study depending on the TCO material and the number of the graphene monolayers transferred.

No. Graphene Monolayers	80 nm-Thick TCO Material	*R_s_* (Ω/sq)	Average White-Light Transmission (%)	FOM (× 10^–^^4^ Ω^–^^1^)
2	AZO	890 ± 70	86 ± 2	2.4 ± 0.9
3	AZO	116 ± 6	80 ± 3	9.2 ± 0.5
1	ITO	72 ± 5	85 ± 2	27.3 ± 0.4
